# Autophagy in the mammalian nervous system: a primer for neuroscientists

**DOI:** 10.1042/NS20180134

**Published:** 2019-09-11

**Authors:** Fumi Suomi, Thomas G. McWilliams

**Affiliations:** 1 Translational Stem Cell Biology and Metabolism Program, Research Programs Unit, Faculty of Medicine, Biomedicum Helsinki, University of Helsinki, Haartmaninkatu 8, Helsinki 00290, Finland; 2 Department of Anatomy, Faculty of Medicine, Biomedicum Helsinki, University of Helsinki, Haartmaninkatu 8, Helsinki 00290, Finland

**Keywords:** autophagy, mitochondria, neurodegeneration, organelles, neurons, metabolism

## Abstract

Autophagy refers to the lysosomal degradation of damaged or superfluous components and is essential for metabolic plasticity and tissue integrity. This evolutionarily conserved process is particularly vital to mammalian post-mitotic cells such as neurons, which face unique logistical challenges and must sustain homoeostasis over decades. Defective autophagy has pathophysiological importance, especially for human neurodegeneration. The present-day definition of autophagy broadly encompasses two distinct yet related phenomena: non-selective and selective autophagy. In this minireview, we focus on established and emerging concepts in the field, paying particular attention to the physiological significance of macroautophagy and the burgeoning world of selective autophagy pathways in the context of the vertebrate nervous system. By highlighting established basics and recent breakthroughs, we aim to provide a useful conceptual framework for neuroscientists interested in autophagy, in addition to autophagy enthusiasts with an eye on the nervous system.

## Introduction: neural complexity and quality control

Membranous organelles make intimate contacts with each other and serve as platforms for multiple molecular processes executed by signalling proteins. This crosstalk occurs with an astounding degree of intracellular dynamism against the backdrop of both phenotypic and environmental context. Amidst this cellular landscape, all components are subject to damage and dysfunction. Accordingly, eukaryotic cells have evolved sophisticated quality control mechanisms to sustain homoeostasis and function. For the human nervous system, sustaining homoeostasis over decades is particularly important. With its trillions of connections, mammalian neural architecture presents a unique physiological conundrum. Neurons are post-mitotic, have high metabolic demands and are often expansive in their morphological configuration and synaptic complexity. Each neural process and projection represents a distinct biochemical compartment, and the seamless interplay of numerous quality control processes is required to safeguard system integrity. To maintain organellar integrity within highly arboreous neurons that project over considerable distances, these quality control pathways are highly dependent upon both intracellular (anterograde, retrograde) and extracellular trafficking mechanisms (transcellular signalling).

One such quality control process is autophagy, a mechanism that evolved to sustain cells during instances of nutrient deprivation [1–3]. The term ‘autophagy’ derives from the Greek ‘*to eat oneself*’, and its use in language dates back to the 1800s [4]. Autophagy is essential for eukaryotic homoeostasis and has profound importance in the nervous system. The basic idea underpinning autophagy is that defective or superfluous subcellular components are sequestered and delivered to the lysosome by various routes for destruction. The autophagic turnover of intracellular components is thought to fuel metabolism.

**Figure 1:**
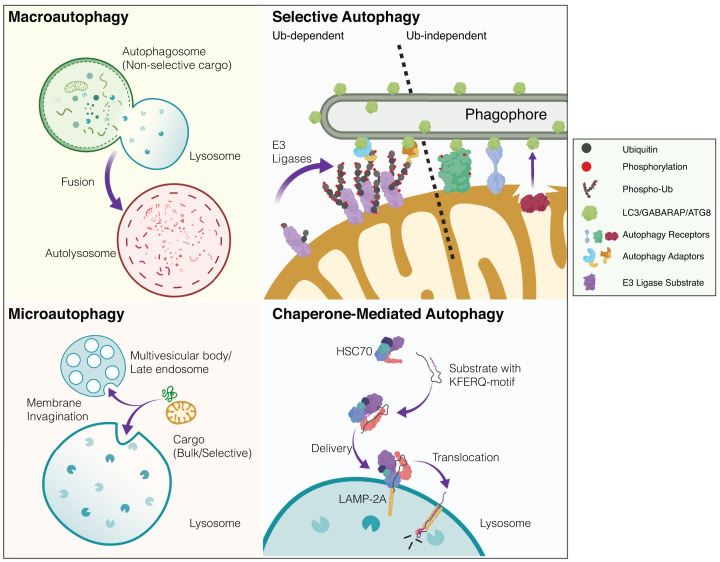
Simplified overview of mammalian autophagy modalities There are several distinct modes of autophagy in eukaryotic cells. These subtypes are defined according to how cargo is recognised and delivered to the acidic or degradative cellular subcompartments (i.e. lysosomes or late endosomes). **Macroautophagy:** cytoplasmic constituents such as organelles and protein aggregates are non-selectively engulfed in a double-membrane bound autophagosome. This bulk sequestration of cellular matter results in degradation upon the formation of the autolysosome (autophagosome-lysosome fusion). The homoeostatic balance between degradation and biosynthesis is ensured by the process of autophagosome-lysosome reformation (not depicted). **Microautophagy:** in contrast with autophagosomal delivery, cargo destined for destruction enters the endolysosomal system *via* membrane invagination. This occurs in non-selective and selective manners with chaperones. Multi-vesicular bodies (MVBs) are also known to participate in microautophagy. **Selective autophagy**: distinct substrates (organelles, proteinaceous/membranous aggregates, pathogens) are recognised *via* priming by cargo-specific receptors. Selective autophagy of organelles (organellophagy) can occur in a ubiquitin-dependent or -independent fashion. The depicted example shows mitophagy where in response to stress, ubiquitin E3 ligases can trigger mitochondrial ubiquitylation, thereby recruiting specific adaptor proteins that engage the autophagy machinery. In ubiquitin-independent selective autophagy, specific receptors directly engage LC3/GABARAP/ATG8 proteins to drive the encapsulation and elimination of the organelle. Lipids such as cardiolipin and ceramide have also been reported as mitophagy receptors (not depicted). Although mitophagy is largely studied as an intracellular process, transcellular mitophagy (known as axonal transmitophagy in neurons) has been described in the CNS (not depicted). Mitochondrial subdomains can also be excised and delivered to the lysosome *via* mitochondrial-derived vesicles (MDVs, not shown). **Chaperone-mediated autophagy (CMA)**: substrate proteins containing a distinct motif are selectively recognised by chaperones and delivered to lysosomes where they undergo translocation and destruction. Created with BioRender.

Several distinct forms of autophagy exist and these are categorised according to how cargo is recognised, processed and delivered to acidic endolysosomal compartments (Figure 1 provides a generalised graphical overview of autophagy modalities). Macroautophagy refers to the non-selective or ‘bulk’ sequestration of cytoplasmic constituents (organelles, protein aggregates, intracellular pathogens) into a transient double-membrane bound autophagosome. The autophagosome eventually fuses with an acidic compartment of the endolysosomal system (autolysosome or amphisome in the case of late endosome–autophagosome fusion), destroying its contents. This pathway contrasts with microautophagy, where cytoplasmic components enter the late endosome or lysosome *via* membrane invagination. Even more distinct than this, chaperone-mediated autophagy (CMA) degrades substrate proteins harbouring specific motifs (typically ‘Lys-Phe-Glu-Arg-Gln’ (KFERQ)-like motif) that are recognised by chaperones and translocated into lysosomes *via* specific membrane receptors [5] (Figure 1). In this brief review, we highlight some essential and emerging concepts in autophagy research, with a particular emphasis on the contribution of autophagy signalling to mammalian neural integrity.

## Autophagy essentials: a generalised overview

Macroautophagy is induced in response to many stimuli (e.g. starvation, hypoxia and infection) and the mechanistic regulation of this process is highly conserved across eukaryotes. These induction signals are often used to study macroautophagy in the laboratory, yet their interplay in modulating macroautophagy *in vivo* remains less clear.

The most upstream signalling component in mammalian autophagy is ULK1, a kinase complex (consisting of ULK1, FIP200, ATG13, ATG101) that initiates autophagosome biogenesis [6]. RNAi-mediated or small molecule inhibition of ULK1 is sufficient to suppress autophagy [7–11]. The ULK1-dependent phosphorylation of downstream substrates (class III PI3K VPS34) stimulates the biosynthesis of phosphatidylinositol-3-phosphate (PI3P) for phagophore nucleation [12,13]. Starvation-induced autophagy requires the VPS34-BECN1 PI-3 kinase and the ULK1 protein kinase for phagophore initiation [14]. Growth factor deprivation and a decreased energy state act as particularly potent induction stimuli for autophagy, with the latter stimulus activating the master metabolic regulator AMPK [15]. The mammalian target of rapamycin (mTOR) is a master nutrient sensor, and active mTORC1 associates with ULK1 [9,16,17]. Reciprocally, mTOR inhibition (either by nutrient deprivation or chemical inhibition) is as a powerful signal that activates macroautophagy [18], leading to its dissociated from, and enhanced kinase activity of ULK1. Independently of its essential role in autophagy, ULK1 has also been implicated in axonal branching [19,20].

The precise source of autophagic membranes has been a hot topic in the field for many years, with many cellular compartments predicted to contribute to precursor membranes. Because of its well-documented association with isolation membranes, subdomains of the endoplasmic reticulum (ER) are regarded to make a crucial contribution to autophagosome formation [21,22]. In addition, ER–Golgi hybrid intermediates, the Golgi complex and mitochondria have also been predicted to contribute to autophagosome biogenesis [23,24]. In recent years, increasing evidence has also mounted in favour of recycling endosomes as a critical source of autophagic membranes [25,26].

The efficiency of autophagosome membrane expansion, maturation and completion involves two ubiquitin conjugation systems: ATG12-ATG5-ATG16L1 and the Ubiquitin-like LC3 subfamily, respectively (also referred to as ATG8-like family). These conjugation systems stimulate the expansion of the autophagosome by promoting the recruitment of small ubiquitin-like ATG8 proteins to membrane-bound phosphatidylethanolamine (PE). These include the LC3 and GABARAP protein subfamilies, which are integral to autophagy. Cleaved LC3-I conjugates with PE, leading to LC3-II formation and ultimately autophagosome maturation [27]. Due to dynamic cycles of synthesis and degradation, LC3 levels fluctuate within cells. Because LC3-II is associated with autophagosomes and accumulates with chemical inhibition (e.g. Bafilomycin A1, chloroquine), it has been used extensively as a biochemical readout of autophagic flux, however there are caveats to this approach [28]. For more in-depth coverage of this highly complex process, the reader is directed to recent reviews [29–32].

Even when mammalian cells have ample access to nutrients, they still recycle damaged or superfluous organelles with exquisite specificity. Distinct from macroautophagy, this is known as ‘selective autophagy’, and is achieved through specialised receptors that confer specificity on the cellular component destined for elimination [33]. Recent *in vivo* research demonstrates that many selective autophagy pathways are likely to operate constitutively within tissues (e.g. mitophagy). However, for reasons of experimental tractability, these pathways have clasically been studied as induced ‘damage responses’ in proliferating cells under conditions of extreme stress, due to the high mechanistic resolution afforded by this approach [29,34]. Sophisticated approaches in mouse genetics and optical reporters now enable researchers to reconcile the *in vivo* significance of *in vitro* mechanisms. Selective autophagy has also been referred to as ‘organellophagy’, and constitutes a burgeoning area of the field [35], where the delineation of multiple selective autophagy pathways continues in ever greater detail.

## Neural macroautophagy in mammalian health and survival

The continual development of facile assays to monitor macroautophagy underpins our present-day knowledge of this process in health and disease [36]. For many decades, researchers relied heavily upon electron microscopy (EM) to visualise morphically distinct autophagic structures in cells and tissues. Indeed, pioneering contributions to histochemistry and EM by Alex B. Novikoff [37] were critical in the early days of the autophagy field and enabled crucial insights by De Duve, Palade and others. Although EM remains the gold standard for high-resolution ultrastructural studies, many proponents recognise its limitations for tissue-based physiological studies. EM is highly labour-intensive, difficult to optimise for high-throughput analyses and requires specialist expertise for execution and accurate interpretation [38]. A pioneering study in 2004 by Noboru Mizushima and colleagues generated a fluorescent autophagy reporter mouse (LC3-GFP), enabling the systemic visualisation of autophagosomes in mammalian organ systems for the first time [39]. This important tool provided a snapshot of steady-state LC3-dependent macroautophagy (hereafter referred to as basal) levels across multiple tissues. The authors conducted extensive validation, showing the pronounced induction of macroautophagy in response to food restriction in mice, and a dramatic reduction in autophagy in *Atg5*-null mice [40]. The autophagic response to starvation differs between organ systems and tissues, indicating the complexity of this process *in vivo*. Originally, CNS neurons were reported to have minimal levels of basal macroautophagy, with reduced autophagy induction in response to starvation. This observation contrasted greatly with other organ systems (e.g. liver, heart, pancreas), where pronounced time-dependent changes in autophagic induction were observed [39]. However, later studies reported a dramatic induction of autophagy in cortical neurons, Purkinje cells and hypothalamic neurons by fasting [41,42].

Classically, macroautophagy serves to sustain metabolism and homoeostasis by ensuring an adequate pool of essential constituents through the catabolism of cellular components. In cultured cells and tissues, nutrient deprivation induces a pronounced macroautophagy response [43]. In terms of timeframe, this response occurs rapidly in cultured adherent cells *in vitro* but requires at least 24 h for a noticeable elevation above basal levels for some mammalian tissues. Although basal macroautophagy in the CNS is reported to occur at low levels and minimally induced upon starvation (compared to other tissues), it has profound importance in the nervous system [39]. This was elegantly demonstrated in two landmark studies that employed conditional genetic ablation of neuronal *Atg5* and *Atg7* (*Atg5^flox/flox^*; *nestin-Cre* and *Atg7^flox/flox^*; *nestin-Cre*) [44,45]. The selective loss of basal macroautophagy in neurons triggered profound phenotypes in these mice, characterised by severe locomotor and gait dysfunction, the intraneuronal accumulation of proteinaceous inclusions, coupled with widespread neuronal loss. In addition to this, mice with Purkinje-neuron-specific deletion of *Atg7* (*Atg7^flox/flox^*; *Pcp2-Cre*), exhibited cell-autonomous dystrophy with terminal axon degeneration [46].

Taken together, this provided a solid physiological basis for basal macroautophagy as a critical modulator of mammalian neural integrity. Furthermore, it provided an essential proof-of-principle for autophagy as a critical mechanism to neutralise aggregate-prone proteins, which are a hallmark of many neurodegenerative diseases. Importantly, the disruption of many discrete steps in the autophagy pathway is associated with the molecular pathophysiology of several neurodegenerative diseases [15,47,48].

Kuma and colleagues [49] also showed the importance of autophagy in the early stages of neonatal life, often termed the ‘starvation phase’. Constitutive autophagy appears to proceed at a low level, with a dramatic, programmed elevation observed during the initial hours of postnatal development [49].

An elegant study by Yoshii and colleagues [50] has recently clarified the physiological significance of neuronal macroautophagy for systemic health and survival. Although a whole-body genetic deletion of *Atg5* results in neonatal lethality, this phenotype can be dramatically rescued by the selective restoration of macroautophagy in neurons (*Atg5*−/−; *Nse-Atg5*). In this remarkable demonstration, these surviving animals have preserved neuronal autophagy, but are deficient in Atg5-dependent macroautophagy in all other extra-neural tissues. Although these animals survive because they can feed, they still exhibit phenotypic abnormalities with hypogonadism and anaemia [50]. Nonetheless, this work demonstrates the importance of neural autophagy for mammalian health and survival.

Although the loss of macroautophagy in the nervous system is clearly deleterious, it is important to recognise that autophagy inhibition represents an advantageous therapeutic strategy in certain contexts [51]. Recently, the systemic inhibition of autophagy had striking anti-tumorigenic effects in a pre-clinical model of *KRAS*-driven lung cancer [52]. However, these benefits were offset by the onset of severe neuropathology, leading to early death in these animals [53]. Conversely, agents that enhance autophagy are considered to have potential to attenuate age-associated cognitive decline. Related to this, autophagy has recently been suggested to promote memory formation by modulating hippocampal-dependent adaptive responses in mice [54]. Enhanced autophagy activation has recently been associated with beneficial cognitive effects in a mouse model of Fragile-X syndrome [55].

Autophagy-related factors also fulfil critical non-canonical (autophagy-independent) roles in the nervous system. For example, ULK1 signalling makes an important autophagy-independent contribution to axonal guidance in mammals [19,20]. Conditional ablation of both *Ulk1/2* in mouse neurons causes selective impairments in axonal tract formation, leading to abnormal forebrain development *in vivo* [20]. Conditional *Ulk1/2* -deficient mice also exhibit a progressive loss of hippocampal CA1 pyramidal neurons at 8 weeks of age [56]. Furthermore, loss of the key ULK1 interactors FIP200 and ATG101 in model organisms affects neural integrity *in vivo*. Conditional loss of FIP200 in the mouse nervous system leads to cerebellar neurodegeneration [57]. Loss of *Drosophila* Atg101 affects neural homoeostasis [58].

## Macroautophagy in mammalian neuropathology

As described in the general overview and elaborated in greater depth in the associated references, macroautophagy is a complicated multistep process. Strikingly, disrupting almost any step in this pathway has disease significance, and dysfunctional autophagy is associated with a diverse range of inherited and idiopathic neurodegenerative disorders [59]. The discovery of ubiquitin/p62-positive intraneuronal aggregates in *Atg7^flox/flox^*; *Nes-Cre* mice, provided an important proof-of-concept demonstration that macroautophagy is a crucial neuroprotective mechanism in mammals [60]. Proteinaceous aggregates are a pathological hallmark of several neurodegenerative diseases (e.g. ‘proteinopathies’, e.g. tauopathies, synucleinopathies depending on the particular disease aetiology, in addition to Huntington’s disease, Amyotrophic Lateral Sclerosis (ALS) and others). Elegant mechanistic work in cultured cells combined with mouse genetics has dramatically expanded this domain – please consult Table 1 for a list of autophagy-associated mouse models with neurological phenotypes. For an excellent disease-specific discussion of evidence implicating autophagy and aggregate clearance in specific neurodegenerative disorders, please consult Menzies and colleagues, 2017 [15]. It is not surprising that autophagy now represents an attractive therapeutic target for neurodegenerative disease and beyond [61].

**Table 1:** Autophagy mouse models and known phenotypes

Gene	Yeast homologue	Model	Phenotype/findings	Reference
***Atg3***	*Atg3*	*Atg3*−/−	Neonatal lethality	[62]
***Atg5***	*Atg5*	*Atg5*−/−	Neonatal lethality	[46]
***Atg5***	*Atg5*	Neural specific *Atg5*−/−	Progressive deficits in motor neurons; accumulation of intraneuronal inclusion bodies	[45,63,64]
***Atg5***	*Atg5*	*Atg5* deletion in POMC neurons	No reported phenotype	[65]
***Atg7***	*Atg7*	*Atg7*−/−	Neonatal lethality	[49]
***Atg7***	*Atg7*	*Atg7 flox/flox*	Liver tumours; neurodegeneration	[63,64,66]
***Atg9a***	Atg9	*Atg9a−/−*	Neonatal lethality	[65]
***Atg12***	*Atg12*	*Atg12-/-*	Neonatal lethality	[67]
***Atg12***	*Atg12*	*Atg12* deletion in POMC neurons	Diet-induced obesity	[67]
***Atg13***	*Atg13*	*Atg13−/−*	Embryonic lethality	[68]
***Atg16L1***	*Atg16*	*Atg16L1* mutant mouse	Neonatal lethality	[44]
***Becn1***	Atg6/Vps30	*Beclin1−/−*	Neonatal lethality	[69–72]
***Gabarap***	*Atg8*	*Gabarap* KO mouse	No reported phenotype	[73]
***Lc3***	*Atg8*	*Lc3*−/−	Normal development	[74]
***Pik3c3/Vps34***	*Vps34*	*Pik3c3/Vps34* specific deletion in sensory neuron	Rapid neurodegeneration	[75]
***Pik3c3/Vps34***	*Vps34*	*Pik3c3/Vps34*−/−	Embryonic lethality	[76]
***Ulk1***	*Atg1*	*Ulk1−/−*	Delayed mitochondrial clearance in reticulocytes	[77]
***Ulk2***	*Atg1*	*Ulk2−/−*	No pheynotype	[78]
***Ulk1/2***	*Atg1*	*Ulk1/2*−/−	Neonatal lethality	[78]
***Ulk1/2***	*Atg1*	*Ulk1/2-cDKO*	Early embryonic and perinatal lethality/abnormal axon guidance	[20]
***FIP200/Rb1cc1***	*Atg17*	*FIP200* ^f/f^ mouse	Cerebellar degeneration	[57]
***Wipi4***	*Atg18*	*Wdr45flox/Y*	Poor motor coordination	[79]
		Specific deletion in CNS	Impaired learning and memory; extensive axon swelling with numerous axonal spheroids	

The recent discovery of human mutations in some core autophagy and autophagy-associated genes has cemented its vital role in neurodegeneration (Table 2). *De novo* mutations in the *WDR45/ATG18* gene are associated with a distinct case of Neurodegeneration with Brain Iron Accumulation (NBIA). This gene encodes WIPI4 and couples PI3P synthesis to LC3 lipidation in autophagosome biogenesis [80]. The clinical features of NBIA include early-onset global developmental delay and progressive neurological deterioration (Parkinsonism, dystonia and dementia developing by early adulthood) [81].

**Table 2:** Disease-associated mutations in human core *ATG* genes

Gene	Yeast homologue	Disease	Reference
***WDR45***	*Atg18/Atg21*	NBIA	[81]
***WDR45***	*Atg18/Atg21*	SENDA (static encephalopathy of childhood with neurodegeneration in adulthood)	[82]
***WDR45***	*Atg18/Atg21*	BPAN (β-propeller protein-associated neurodegeneration)	[83,84]
***ATG5***	*Atg5*	Congenital ataxia, mental detardation, developmental delay	[85]

Additionally, the manifestation of static encephalopathy of childhood with neurodegeneration in adulthood (SENDA) is associated with human mutations in *WDR45*. SENDA is a variant of NBIA reported by Kruer and colleagues in 2012 [82], with affected individuals exhibiting childhood intellectual impairments and severe dystonia-Parkinsonism in adulthood. Patient-derived cell lines exhibited lower autophagic activity combined with the accumulation of aberrant early autophagic structures [83]. SENDA is also known as *β*-propeller protein-associated neurodegeneration (BPAN), and a patient is reported to carry *de novo* heterozygous splice-site mutations of *WDR45* [84]. *Nes-Wdr45 ^fl/Y^* mice exhibit motor coordination and learning defects, accompanied by axon swelling [79].

Regarding the core ATG machinery, the first mutation in human *ATG5* was recently reported. Two childhood siblings presented with cerebellar ataxia, hypoplasia and lack of coordination. These neurological findings were the result of a point mutation in human *ATG5* (E122D), leading to a weak ATG5–ATG12 interaction [85]. As diagnostic resolution continues to advance in clinical genetics, it will be exciting to determine the exact prevalence of patients with autophagy-associated mutations and neurological symptoms.

## The emerging importance of selective autophagy in mammalian neural integrity

Although we have a vast knowledge about the molecular regulation and physiological significance of macroautophagy, selective autophagy pathways have emerged as crucial pathways in their own right [27,33,34,86]. Selective autophagy refers to the targeted delivery of defective or superfluous organelles to the lysosome for elimination and typically operates in ubiquitin-dependent or -independent fashion. In ubiquitin-dependent turnover, cellular sensing of defective organelles/components activates specific ubiquitin ligase enzymes that decorate cargo with ubiquitin chains of varying topologies. This ‘organellar ubiquitylation’ can serve as a distinct ‘eat-me’ signal that engages specific autophagy adaptor proteins, resulting in the recruitment of the autophagy machinery and elimination of the organelle. The number of selective autophagy receptors has expanded dramatically in recent years (detailed in Table 3). For ubiquitin-dependent selective autophagy, deubiquitylases (DUBs) are potent regulators that oppose this process.

**Table 3:** Mammalian selective autophagy receptors

Autophagosomal substrate	Receptors
**Mitochondria/mitophagy**	NIX/BNIP3 [87], FUNDC1 [88], p62 [89], HDAC6 [90], OPTN1 [91], NDP52 [92], NIPSNAP [93], PHB2 [94], FKBP8 [95], Cardiolipin [96]
**Peroxysomes/pexophagy**	P62 [97,98], NBR1 [97], PEX3 [99], PEX2 [100], PEX5 [101], ACBD5(Atg37) [102]
**Ribosomes/ribophagy**	NUFIP1 (Nuclear fragile X-mental retardation-interacting protein 1) [103]
**Lipid droplets/lipophagy**	Macroautophagy/no receptors are identified [104,105]
**Protein aggregates/aggrephagy**	P62 [106], NBR1 [107], ALFY [107,108], OPTN [106], HDAC6 [109], BAG3 [110], NDP52 [111]

Like macroautophagy, which occurs at basal levels and can also be induced by specific stimuli, selective autophagy pathways also occur at basal levels and can be induced in response to stress. The following section provides some historical context, and attempts to elaborate the most recent concepts in this field. We pay particular attention to mitochondrial turnover, given the significant advances made in this domain.

### Mitophagy

#### Mitophagy in healthy tissues

The turnover of mitochondria (termed mitophagy) is arguably the most intensively studied pathway in the selective autophagy field. Mitochondria are dynamic signalling organelles that orchestrate myriad aspects of mammalian metabolism from energetics to epigenetics. Beyond ATP production and burning fuel, these pleiotropic powerhouses contribute to the biosynthesis of nucleotides, amino acids, haem, Fe–S clusters, sterols, as well as playing critical roles in organelle biogenesis and calcium homoeostasis [112]. Consequently, mitochondrial dysfunction is associated with a plethora of neurodegenerative disease states [34,113–115].

Despite the modern-day limelight it receives, it seems that mitochondrial turnover has captured the imagination of researchers for decades. At the time of writing this review, the first known reference to mitochondrial turnover dates to the pioneering work of Margaret Reed Lewis and Warren Lewis, in their 1915 cell biology masterpiece which examined mitochondrial dynamics using Janus green dye in tissue culture experiments [116]. They observed that under certain circumstances, mitochondria can ‘degenerate’ [116]. Interestingly, the term ‘mitochondrial degeneration’ appears again in a diabetes-associated case report from Edinburgh in 1928, but there is insufficient detail to provide a context for any reference to selective elimination [117]. Subsequently, important EM studies in the 1950s by Sam Clark Jr. [118] resolved mitochondria within endolysosomal structures in various mammalian tissues. The first direct paper on the selective elimination of mitochondria appears to come from Novikoff in 1962, in his original study of mitochondrial degeneration [119]. It is of interest that 6 years earlier, Novikoff also identified ‘dense bodies’, which linked degradative cellular compartments with mitochondria and other organelles [120]. The reader should note that in all of these aforementioned investigations, researchers were studying basal levels of mitochondrial turnover (often in healthy wild-type, unstressed animals), which precedes the present-day focus on stress-induced mitophagy in cell culture.

Akin to macroautophagy, it was challenging to observe mitophagy in cells and tissues until the advent of fluorescent reporter mice (these experimental challenges are outlined in [38]). Recent studies characterising the mito-Keima and *mito*-QC mitophagy reporter mouse models have been instrumental in illuminating mitochondrial turnover *in vivo* [121,122]. For an excellent review of reporter mice and physiological autophagy, please consult Kuma and colleagues [40]. The mitochondrial matrix localisation of mt-Keima and mitochondrial outer membrane localisation of *mito-*QC demonstrate an abundance of mitochondria in lysosomes (mitolysosomes) across a variety of organ systems) [121–125]. Importantly, both reporter models provide an unambiguous demonstration that mitophagy occurs at steady-state levels within mammalian tissues. In terms of mitochondrial turnover in the vertebrate nervous system, studies in *mito*-QC mice have illuminated the spatial nature of mitophagy within a variety of neural subtypes *in vivo*. These comparative investigations revealed a robust level of mitophagy in A9 dopaminergic neurons and cerebellar Purkinje neurons, with a striking spatial restriction of turnover to neuronal somata [122–124]. Additionally, microglia and cerebral vasculature also had noticeably high levels of mitochondrial turnover [122]. Recent data utilising these reporters also demonstrated the conservation of basal mitophagy from mammals to flies [126,127].

Mitophagy has also been shown to contribute to CNS development. Elegant work by Boya and colleagues demonstrated the importance of mitophagy in retinal neurodevelopment during the metabolic transition towards glycolysis [128]. Furthermore, high levels of basal mitophagy have also been observed in photoreceptor neurons [129].

An important concept that is pertinent to the complexity of the mammalian nervous system is that the process of mitophagy may not always be intracellular. Davis and colleagues [130] used a mitophagy reporter combined with EM analyses to demonstrate the extrusion of mitochondria from retinal ganglion axonal evulsions, and degraded by lysosomes within neighbouring astrocytes. Interestingly, this atypical mitophagy modality termed as ‘axonal transmitophagy’ was observed to occur under steady-state conditions [130]. Conversely, neurons have also been described to acquire mitochondria from glia following ischaemic injury [131]. It is tempting to speculate that such mechanisms may facilitate long range homeostasis in the vertebrate nervous system. Collectively, these studies highlight that much more work is required to decipher the differential mechanisms and significance of neural mitophagy *in vivo*.

#### Mitophagy in neuropathology

Mitochondrial and neural integrity are intimately linked. In particular, generations of researchers have sought to clarify the mysterious relationship between mitochondrial and motor dysfunction in the context of Parkinson’s disease (PD). The first known studies of mitochondrial integrity in PD-patient biospecimens emerged from two reports in the 1970s, from Japan and Sweden, respectively [132,133]. Although severely limited in both scope and resolution, these early papers postulate a conceptual link between mitochondrial function and Parkinsonism [133]. Relevant mechanistic links emerged from pioneering work by Langston and colleagues [134] who discovered that the Parkinsonian neurotoxin MPTP exhibits a mitochondrial mode of action. In the late 1980s, reduced mitochondrial activity was also observed in PD patient brain-specimens [135], although it remains contentious whether or not this is a generalised feature of Parkinsonism [136]. A direct genetic link between mitochondrial dysfunction and PD emerged in 2004 by Suomalainen and colleagues, who discovered L-DOPA responsive Parkinsonism in patients with defective mtDNA maintenance, conferred by mutations in *POLG* [137].

Because the cause(s) of idiopathic PD remain elusive, rare inherited forms of familial Parkinsonism have provided important molecular insights into potential mechanisms of pathophysiology. Many of the numerous loci that are causative for EOPD (early-onset PD) influence mitochondrial signalling [115]. Present-day interest in mitophagy stems from the vital observation that two of these PD-related proteins, PINK1 (encoded by *PARK6*) and Parkin (encoded by *PARK2*) can orchestrate the dramatic clearance of mitochondria in cultured proliferating cells under highly defined conditions. This pathway has been reviewed extensively in the literature (reviewed in [34,114,115,138]).

Briefly, PINK1 is a mitochondrial-associated ubiquitin kinase [139], and Parkin is a cytosolic RBR E3 ubiquitin ligase that exists in an inactive (autoinhibited) state. Upon loss of mitochondrial membrane potential, PINK1 becomes stabilised and activated on depolarised mitochondria, and Parkin is recruited to the damaged organelle. PINK1 then phosphorylates both Parkin and ubiquitin at their respective Ser^65^ residues. The binding of phospho-Ser^65^-ubiquitin to Parkin, primes it for full activation by PINK1 at Parkin^Ser65^. These events drive a feed-forward activation loop of mitochondrial ubiquitylation, whereby damaged mitochondria become decorated in a coat of ubiquitin and selectively eliminated *via* autophagy (reviewed in [114,115,140]).

Aside from Parkin, other factors have been implicated in mitochondrial ubiquitylation, including ARIH1, FBX07, Gp78, MARCH5, MUL1, RNF5, RNF185, SIAH1 and SMURF1 [34,114]. Yun and colleagues discovered an exciting compensatory role for MUL1 (also known as MULAN and MAPL) [141,142] during the loss of PINK1/Parkin signalling in flies and mice [143]. Lastly, DUBs appear to be critical negative regulators of mitophagy *in vitro*, but also influence pexophagy [144]. Bingol and colleagues first reported that USP30 antagonises Parkin-mediated mitophagy [145]. The contribution of USP30 to mitophagy in mammalian tissues remains to be clarified, and the analysis of mitophagy in *Usp30* mutant animals will surely prove interesting.

Mitophagy is not just a ubiquitin-dependent process and as such, many ubiquitin-independent mitophagy receptors have also been identified. These include FUNDC1, NIX/BNIP3L, NIPSNAP, PHB2, FKBP8 and Cardiolipin (Table 3) [34,114]. Aside from mitochondrial depolarisation, PINK1/Parkin-independent mitophagy can be robustly induced by iron chelation and hypoxia [114,115] For a recent and authoritative overview of these pathways, please consult Montava-Garriga and Ganley [146].

#### A provocative reassessment of mitophagy *in vivo*

It has been challenging to assess the contribution of mitophagy to adult neural integrity, because we do not yet know of any candidate genes that modulate the selective, steady-state autophagy of mitochondria *in vivo*, i.e. mitophagy-deficient mammals are yet to be described. Genetics studies have demonstrated that macroautophagy-deficient animals exhibit developmental lethality [45,46,62–64] (Table 1). Thus, it seems reasonable to assume that animals unable to neutralise mitochondrial damage *via* mitophagy would be equally compromised, or at least exhibit phenotypic severity from an early age. This is particularly relevant given the importance of programmed mitophagy in CNS development [125,128]. Furthermore, given the importance ascribed to mitochondrial quality control in sustaining tissue homoeostasis [112,147], is it likely that a mitophagy-deficient animal would even survive?

As previously discussed in this review, numerous historical studies have documented mitochondria within degradative cellular compartments, yet recent research on basal mitophagy has provoked some level of controversy in the field [34,140,148]. Why has such a contentious debate ensued? Over the past decade, many reviews and research papers have cited the PINK1-Parkin signalling pathway to be a ‘master regulator’ of mitophagy, and posit defective mitophagy to be the elusive cause of PD. Although this was an intriguing hypothesis and is still regarded by some as an attractive therapeutic target for PD, the regulation of mitophagy *in vivo* appears to be far more complicated than cell culture models would predict. Furthermore, there are notable caveats that have been well described with respect to PINK1/Parkin-dependent mitophagy [27,34,114,126]. For example, the induction of PINK1/Parkin-dependent mitophagy in proliferating cancer cells requires the use of toxic agents (e.g. protonophores such as CCCP) in concert with extremely high levels of Parkin expression. Recently, the activation of depolarisation-induced mitophagy was shown to be affected by the concentration of serum components in tissue culture media, specifically bovine serum albumin [149]. Until recently, there was a lack of suitable biochemical tools to detect endogenous PINK1 protein in mouse tissues under stringent conditions (i.e. verifying the specificity of antibody reagents with suitable KO controls and verification by mass spectrometry [124]). Despite this, it is noteworthy that the endogenous signalling pathway can be activated in cultured primary neurons, although neural mitophagy was unaffected in both mice and primary PD-patient cells lacking a functional PINK1/Parkin pathway [123].

*Pink1* and *Parkin* mutant mice do not recapitulate the authentic hallmarks of human PD [150], and substantial levels of genetic and mitotoxic stress are often required to elicit measurable phenotypes in KO animals (although recent knock-in strategies have resulted in selective phenotypes [123]). Even though these interventions lead to motor dysfunction and neurodegeneration in some animals, they are not accompanied by alterations in nigrostriatal mitophagy [151,152]. Furthermore, several converging studies now show that basal mitophagy proceeds unhindered in tissues from both *Pink1* KO animals and *Parkin* mutant mice with loss of E3 enzyme activity [123,129]. These observations of PINK1-Parkin independent mitochondrial turnover have been replicated in flies, mice, non-human primates and cell culture systems [126,153–155]. Intriguingly, although stress is required to recapitulate relevant PD phenotypes in adult *Pink1/Parkin* KO mice, this is not the case with rat and non-human primate models [156–158]. Thus, further work will be needed to clarify the significance of these differential phenotypes that manifest in a species-specific manner.

The manifestation of neuropathology in familial PD has informative distinctions from the idiopathic condition. Intracellular inclusions known as Lewy bodies were first described in 1919 by Konstantin Tretiakoff and are regarded as a classical hallmark of PD pathology. Indeed, recent ultrastructural studies of postmortem Lewy body composition from human PD patients underscore the importance of macroautophagy and organellar integrity in the nervous system [159]. Despite this, inclusion pathology is not a consistent feature of PINK1/Parkin-associated PD in mouse models or patients (some Lewy pathology has been documented in case reports from *PINK1* and *PARKIN* patients, although this appears to be extremely rare) [160]. These differential aetiologies merit consideration, especially when considering defective mitophagy as the sole pathological cause of any complex disease.

Because basal mitophagy is not affected in animals lacking a functional PINK1-Parkin pathway, does this imply that the PINK1-Parkin signalling is completely irrelevant for mitophagy? We do not regard this assertion to be constructive or accurate, especially given the complexity of signalling in the context of the mammalian nervous system. Mutations in both PINK1 (*PARK6*) and Parkin (*PARK2*) lead to early-onset PD. Furthermore, the recent discovery of a new human PD mutation at Parkin S65 (S65N) demonstrates that loss of PINK1-dependent Parkin activation compromises striatal and motor function in mice and humans [123]. Under basal conditions, mitophagy was unaffected in mouse tissues and patient-derived cells with mutant Parkin^Ser65^. The fact that mitophagy can proceed in the absence of functional PINK1-Parkin signalling, demonstrates that mammalian tissues require more than a single pathway to modulate mitochondrial turnover. In addition to its association with PD, mitophagy has also been implicated other neurodegenerative disease states. A recent study by Fang and colleagues [161] has implicated defective mitophagy in the pathophysiology of Alzheimer’s disease (AD).

Our view is that mitophagy is a context-dependent process, consisting of distinct basal and stress-induced pathways. This nuanced perspective is consistent with the growing consensus espoused in recent comprehensive reviews on this topic [34,121]. At present, the tissue-specific factors that modulate basal mitophagy *in vivo* remain to be defined. However, the most physiological regulators of steady-state mitophagy described to date appear to be NIX/BNIP3L [87], ULK1 and p62 [89]. Mutant *Nix* or *Ulk* animals exhibit an accumulation of abnormal mitochondria within reticulocytes *in vivo* (Table 3), and p62 is required for hepatic mitophagy [155].

#### PINK1/Parkin in vivo: if not mitophagy, then what?

Even if the loss of PINK1/Parkin does not influence steady-state mitophagy *in vivo*, the requirement of this pathway to sustain homoeostasis during profound levels of cellular stress is exceptionally intriguing. Thus, a key question remains: what is the mechanistic contribution of this pathway to neural integrity?

Rather than influencing steady-state mitochondrial turnover *in vivo*, it is conceivable that PINK1 and Parkin could orchestrate mitophagy/another form of mitochondrial quality control during highly defined conditions of extreme stress. In line with this, *Pink1/Parkin* KO mice crossed with mitochondrial mutator *Polg*^D257A^ mice and subjected to exhaustive exercise, a STING-mediated inflammatory response accompanied the onset of neurodegenerative phenotypes [152]. Although there were no reported differences in basal mitophagy between unstressed *Pink1* mutant animals, this intriguing experimental paradigm revealed defective cardiac mitophagy in aged mutant animals subjected to extreme stress, with circulating mtDNA driving a neuroinflammatory response [152]. Despite a reported mitophagy defect in the hearts of double-mutant animals (*Pink1* or *Parkin* KO mice crossed to *Polg*^D257A^ mutator mice, aged and subjected to exhaustive exercise) it is unclear whether mitophagy was affected in the PD-relevant nigrostriatal pathway. It is also interesting to note that mass spectrometry analysis revealed minimal differences in phospho-ubiquitin levels between WT and *Pink1* KO animals at steady-state levels. These emerging links between mitochondrial and immune integrity are especially interesting. A 2014 study from the laboratory of Mike Murphy discovered that *Polg*^D257A^ mice have elevated levels of circulating pro-inflammatory cytokines upon ageing (TNF-*α*, IL-1*β*) [162]. Yet, a comparative analysis of serum cytokine profiles between the uncrossed mutator mice in the Sliter and colleagues study suggests a very different immunological profile. Nonetheless, it will be intriguing to interrogate these findings and dissociate the mechanistic contribution of PINK1-Parkin signalling from the already complex background of mutator mice.

Recent *Drosophila* studies also support the notion that PINK1-Parkin signalling is dispensable for basal mitophagy, but may also play a role in stress-induced mitophagy [126,127]. Independent of mitophagy, other studies have delineated the mechanics of how matrix-localised mtDNA can activate cGAS-STING signalling [163], and have also linked cGAS-STING signalling to macroautophagy [164].

It is important to note that PINK1 and Parkin have also been implicated in the formation of ‘mitochondrial-derived vesicles’ (MDVs), a discrete form of quality control, whereby defective mitochondrial components or cargo-specific regions undergo a process of (1) budding (2) excision from mitochondria and (3) delivery to the endolysosomal system for elimination (reviewed in [165]). Although mitophagy and the MDV pathway ultimately have the same terminal destination (the lysosome), the cargo-specific nature of the MDV pathway represents a more targeted variant of quality control. It will be exciting to unearth the spatial regulation of MDVs *in vivo*, as has been done for mitophagy and macroautophagy. The PD-associated genes *VPS35, PINK1* and *PARKIN* have been linked to the generation of particular MDV subsets.

These exciting links between mitochondrial integrity, immunity and PD are also consistent with pioneering collaborative research from the laboratories of Heidi McBride and Michel Desjardins, who discovered the phenomenon of mitochondrial antigen presentation (MitAP) in 2016 [166]. In the absence of PINK1-Parkin signalling, a pro-inflammatory stimulus (*via* lipopolysaccharide treatment) promoted the formation of MDVs and mitochondrial antigens on MHC-I molecules at the surface of antigen-presenting cells [161]. Although these findings suggested an autoimmune component to PD, it remained unclear how to reconcile this with both mitochondrial and nigrostriatal integrity, the latter of which are largely unperturbed in most PINK1/Parkin mutant mice.

Building on their previous discovery of MitAP and independent converging studies that demonstrate PINK1 is dispensable for basal mitophagy *in vivo*, a recent landmark study from Matheoud and colleagues [167] clarifies the contribution of both MitAP and PINK1-Parkin signalling to striatal integrity *in vivo*. By subjecting *Pink1* KO mice to intestinal infection, they observed the initiation of MitAP and the induction of cytotoxic cellular subset: mitochondrial-specific CD8^+^ T cells [87]. Strikingly, this was accompanied by PD-relevant phenotypes (loss of striatal arborisation and L-DOPA responsive motor dysfunction). Although it remains unclear if these cytotoxic T cells directly drive the degeneration of dopaminergic neurons, these findings both extend our understanding of MitAP and the mitophagy-independent function of PINK1-Parkin signalling in the modulation of adaptive immunity. This paradigm is particularly noteworthy in pre-clinical terms, as mitochondrial stress was not required for the onset of neurological phenoytpes in *Pink1* KO mice. The collective resolution of these experimental stress paradigms (infection, extreme stress) to the onset of human neurodegeneration constitutes an exciting area of future research.

It will also be crucial to expand upon these emergent mitophagy-independent functions of PINK1-Parkin signalling, and clarify their cell-specific roles *in vivo* [34,147]. This line of enquiry will be particularly important, as the manifestation of neuropathology in PINK1/Parkin PD patients is distinct from idiopathic PD cases. As other EOPD loci are known to influence cellular trafficking and autophagy (e.g. LRRK2), it will be exciting to decipher the interplay of these additional PD-related factors to mitochondrial quality control *in vivo*.

#### Could mitophagy therapeutics halt neurodegeneration?

Mitochondrial signalling has emerged as an attractive drug development area [168], and the search for small molecule compounds that could modulate mitophagy in tissues is an important domain of research. In a small-scale human trial, the metabolite Urolithin A (UA) was well tolerated and exerted beneficial effects on mitochondrial metabolism [169]. Although UA modulates mitophagy in *Caenorhabditis elegans* and influences mitochondrial function in rodent models [170], it remains to be determined how UA administration influences mitophagy signalling in human tissues at steady-state levels.

Because no disease-modifying therapies exist to halt PD progression, the PINK1-Parkin pathway has attracted considerable attention as a prospective translational target for PD [140,171]. Recent advances in understanding the atomic structure of PINK1 will surely provide a much-needed blueprint for these efforts [140,144,172]. Although targeting this particular pathway may indeed provide a meaningful benefit for a subset of PD patients, much pre-clinical work will be required to relate these effects to nigrostriatal mitophagy *in vivo*. The continual refinement of new tools to monitor selective autophagy in tissues will surely help to facilitate this [40,125].

Our perspective is that mitophagy-related drug discovery efforts should not hinge on a single signalling pathway. Thus, any anti-climactic or adverse outcomes from any PINK1/Parkin-associated therapeutic strategy should not be conflated with a therapeutic setback for mitophagy. Exciting new data by Matheoud and colleagues demonstrate the mitophagy-independent, physiologically relevant consequences of *Pink1* ablation should merit serious consideration for those interested in targeting this pathway for PD.

Our evidence-based interpretation of the available data is that different physiological contexts necessitate different modalities and mechanisms of mitochondrial turnover [34,114,147]. Depending on when, where and how mitochondrial networks become compromised, our cells have devised more than a single mechanism to destroy imperfect powerhouses. This perspective does not conflict with previous findings but instead seeks to integrate our current understanding of mitophagy into a constructive framework that can drive meaningful translational benefit. It is widely accepted that proliferating cancer cells employ myriad molecular strategies to assert their pathological supremacy [173]. Are the elusive degenerative mechanisms of the mammalian nervous system likely to be any less complex?

Our concerns stem from the sobering state of drug discovery in neurodegenerative disease. A 2018 study reported a 99.6% failure rate for AD drug development with a further significant setback announced this year [174,175]. For neurodegeneration to be ‘the next oncology’ [176] and for PD to escape the same fate of AD, a different strategy will be required. We predict that rigorous efforts to understand the interplay of autophagy signalling pathways and their contribution to neural integrity *in vivo* will be vital in this area. Our perspective is consistent with established leaders calling attention to the multifaceted nature of neurodegenerative pathology [177].

### Pexophagy

Pexophagy refers to the selective elimination of defective or damaged peroxisomes by autophagy and is receiving increased attention in the field. Peroxisomes are single-membrane bound organelles that fulfil vital metabolic functions in lipid, polyamine and amino acid catabolism, lipid biosynthesis, glyoxylate detoxification and ROS/NOS signalling [178]. Mitochondrial and peroxisomal biology are more intertwined than previously realised, illustrated by the striking discovery that mitochondria can generate newly born peroxisomes [179]. Defective peroxisomes are detrimental to nervous system function, illustrated by the many neurological phenotypes with disease mutations in peroxisome-associated genes. For example, the peroxisome biogenesis disorder (PBD) Zellweger syndrome (ZS) is a neurodevelopmental disease [180] characterised by the absence of peroxisomes, profound metabolic deficiencies, in addition to hypomyelination and sensineuronal degeneration. For a comprehensive review on peroxisomal function in health and disease, please consult Cho and colleagues [181].

Interestingly, the PBD-associated genes *PEX2* [182] and *PEX3* [183] encode mammalian pexophagy receptors. Mutant *PEX2* is linked with Infertile Refsum disease (IRD) in humans [182]. Patients with IRD exhibit severe cognitive and behavioural impairments as well as subtle craniofacial abnormalities and retinitis pigmentosa.

Studies in cultured human cells have demonstrated the importance of *PEX2* in modulating mammalian pexophagy [100]. In addition to pexophagy, PEX2 appears necessary for both starvation-induced and constitutive macroautophagy [100]. Genetic ablation of *Pex2* in mice results in a dramatic loss of neuronal arborisation in cerebellar Purkinje neurons *in vivo* [184]. Studies have also linked PEX3 to peroxisome-ubiquitination-linked pexophagy, and NBR1 appears essential for PEX3-dependent pexophagy in cultured HeLa cells [99]. Homozygous mutations in *PEX3* are also associated with severe cases of ZS [183]. The PEX5 peroxisome import receptor is reported to interact with mutated in ataxia telangictasia (ATM), a DNA-damage response-associated kinase, and activated ATM directs ULK1 to induce pexophagy [101]. ATM kinase plays an essential role in cellular homoeostasis, and mutations in *ATM* mutations cause neurodegeneration [185].

The Acyl-CoA binding domain containing protein5 (ACBD5) is a human orthologue of Atg35 and is also associated with pexophagy [186,102]. A pathogenic patient mutation in *ACBD5* is associated with impaired fatty acid metabolism with retinal dystrophy and white matter disease [102].

Recently, the autophagy receptor NBR1 was discovered to be a pexophagy receptor [97]. Loss of endogenous NBR1 triggers an increase in catalase levels, and NBR1 overexpression targets peroxisomes to lysosomes, demonstrating a role for this protein in promoting constitutive pexophagy [97]. Interestingly, NBR1 is regarded to influence the formation of proteinaceous inclusions associated with neuropathology in PD, dementia and multiple system atrophy [187].

As previously mentioned, USP30 is a DUB that modulates Parkin-dependent mitochondrial clearance in cultured cells [188], yet its contribution to mitophagy *in vivo* remains to be determined. In a surprising twist, an elegant study from the Clague and Urbé laboratories recently demonstrated that USP30 associates with peroxisomes and modulates basal pexophagy [154,189].

### Ribophagy

Research in yeast and plants has provided crucial insights into the selective autophagy of ribosomes, and the first demonstration of mammalian ribophagy was made recently in cultured cells using a Ribo-Keima flux reporter [190]. Like most autophagy pathways in cultured cells, basal ribophagic flux occurs at extremely low levels (<1% of ribosomes over a 24-h period). This increases when classical macroautophagy stimuli are applied, i.e. nutrient deprivation and mTOR inhibition promote VPS34-dependent ribophagic flux (∼10% of ribosomes over a 24-h period in human cells). Proteostatic stress using AS (arsenite) was also found to promote ATG5-dependent ribophagic flux.

Recently, an exciting study identified Nuclear fragile X-mental retardation-interacting protein 1 (NUFIP1) as a putative receptor for starvation-induced ribophagy [103]. NUFIP1 interacts with ribosomes and binds LC3B, promoting their autophagic delivery. Conversely, mammalian cells expressing mutant NUFIP1 (that cannot bind LC3B) exhibit impaired ribophagy.

The quality control of ribosomes is known to be maintained by a surveillance mechanism called ribosome-associated protein quality control (ROC) [191], and involves the E3 ubiquitin ligase Ltn1 Mutations in *Ltn1* is reported to cause neurodegeneration in mice [191,192]. Given the spatial nature of translation in neurons [193], it will be certainly interesting to ascertain the role of ribophagy in sustaining neural integrity.

### Aggrephagy

Aggrephagy refers to the selective elimination of proteinaceous aggregates, i.e. aberrantly folded ubiquitin-positive inclusions. Although neuronal protein aggregation occurs during healthy ageing, excessive accumulation is a hallmark of neurodegenerative pathology, including Lewy bodies in idiopathic PD, neurofibrillary τ tangles and amyloid plaques in AD, polyglutamine aggregates in Huntington’s disease [59,194] and ubiquitin/p62 positive inclusions in ALS. Several aggrephagy receptors have been identified (Table 3), and enhancing levels of aggrephagy has evolved as an attractive neuroprotective strategy with therapeutic promise. Interestingly, mutations in many aggrephagy receptors result in neurodegeneration: mutations in *ALFY* are associated with human microcephaly [59,194], *OPTN1* for ALS and primary open angle glaucoma [195], in addition to p62 (*SQSTM1*) mutations which are associated with ALS, frontotemporal dementia (FTD) and distal myopathy [196,197].

## Concluding remarks

Macroautophagy and selective autophagy are fundamental quality control mechanisms with demonstrable relevance for neural integrity. Their contribution to cell type-specific homoeostasis is only beginning to be elucidated, especially in the mammalian nervous system.

It is clear from the classical and contemporary data that a symphony of basal and stress-evoked pathways orchestrate cellular integrity. Further research will be vital to uncover how these pathways become activated *in vivo* and how they converge to sustain neural integrity during a lifetime of varying physiological contexts.

## Abbreviations

ACBD5: Acyl-CoA binding domain containing protein5
AD: Alzheimer’s disease
ALS: amyotrophic lateral sclerosis
ATM: mutated in ataxia telangictasia
DUB: deubiquitylase
EM: electron microscopy
EOPD: early-onset Parkinson’s disease
ER: endoplasmic reticulum
IRD: infertile Refsum disease
MDV: mitochondrial-derived vesicle
MitAP: mitochondrial antigen presentation
mTOR: mammalian target of rapamycin
NBIA: Neurodegeneration with Brain Iron Accumulation
NUFIP1: nuclear fragile X-mental retardation-interacting protein 1
PBD: peroxisome biogenesis disorder
PD: Parkinson’s disease
PE: phosphatidylethanolamine
PI3P: phosphatidylinositol-3-phosphate
SENDA: static encephalopathy of childhood with neurodegeneration in adulthood
UA: Urolithin A
ZS: Zellweger syndrome
KO: Knockout (genetic ablation)
ATG: Autophagy-related
ULK1: Unc-51 like autophagy activating kinase 1 (Yeast Atg1)
LC3: Microtubule-associated protein light chain 3
CNS: Central Nervous System
